# Intratumoral microbiome: a crucial regulating factor in development and progression of colorectal cancer

**DOI:** 10.1186/s43556-025-00376-2

**Published:** 2025-12-15

**Authors:** Yating Fan, Xiangshuai Gu, Hua Yang, Ye Chen, Chao Fang, Hongxin Deng, Lei Dai

**Affiliations:** 1 State Key Laboratory of Biotherapy and Cancer Center, West China Hospital, Sichuan University and Collaborative Innovation Center for Biotherapy, Chengdu, Sichuan 610041 China; 2 Huidong General Surgery Department of Zigong Fourth People’s Hospita, Zigong, 643000 China; 3 Colorectal Cancer Center, West China Hospital, Sichuan University, Chengdu, Sichuan 610041 China

**Keywords:** Colorectal cancer, Intratumoral microbiome, Tumor microenvironment, Immune modulation, Engineered bacteria, Biomarkers

## Abstract

Colorectal cancer (CRC) is one of the most malignant cancers, and studies have indicated that microbes within tumors play a crucial role in CRC. Advanced methodologies, including single-cell and spatial technologies, high-resolution sequencing, and multi-omic integration, are now unraveling the complex composition and function of the intratumoral microbiome. Mechanistically, these microbial communities contribute to CRC initiation by serving as direct mutagens that induce genomic instability, perpetuating a state of chronic inflammation, and activating specific carcinogenic pathways. Furthermore, they actively promote tumor progression and metastatic dissemination through multiple means, including the modulation of key oncogenic signaling pathways, extensive remodeling of the tumor immune microenvironment, and facilitation of a pro-metastatic niche. Given these profound and multifaceted influences, the intratumoral microbiome shows significant promise as a source of diagnostic and prognostic biomarkers, offering considerable potential for non-invasive monitoring and improved risk stratification in clinical practice. Therapeutically, intervention strategies are rapidly evolving, encompassing approaches such as microbiome modulation to enhance conventional therapies, precise clearance of pathogenic bacteria, utilization of intrinsically antitumor microbes, and the engineering of synthetic bacteria as targeted living therapeutics. This review comprehensively outlines the current research methods, elaborates on the mechanistic insights, and discusses the therapeutic targeting of the intratumoral microbiome, aiming to provide a foundational framework for developing new and effective strategies in CRC precision medicine.

## Introduction

Colorectal cancer (CRC) persists as a formidable global health challenge, ranking third in incidence and second in mortality worldwide [1]. Its pathogenesis has traditionally been ascribed to the accumulation of genetic alterations and environmental influences [2, 3]. However, the pervasive influence of the human microbiota has emerged as a critical third dimension in oncogenesis. The gastrointestinal tract, host to a complex ecosystem of trillions of microorganisms, engages in a dynamic symbiosis that is essential for maintaining host homeostasis [4]. A compelling body of evidence now underscores that this microbial influence extends beyond the gut lumen and into the very fabric of tumors themselves [5–7]. Advances in high-throughput sequencing technologies (e.g.,16S rRNA and metagenomic sequencing [8, 9]) have enabled the identification of unique microbial communities within the tumor microenvironment (TME)—termed the intratumoral microbiome. Although characterized by extremely low biomass [10, 11], these microbes exhibit spatial heterogeneity (e.g., differences between cancerous and normal tissues) and functional specificity (e.g., the anti-CRC effects of Lachnospiraceae [12]), offering novel insights into CRC mechanisms.

The intratumoral microbiome shapes tumor evolution through mechanisms such as direct genotoxicity and immunomodulation. Bacterial toxins, including colibactin and Bacteroides fragilis toxin (BFT), directly induce DNA damage and genomic instability. Concurrently, microbial activation of pattern recognition receptors instigates chronic inflammation, thereby fostering a permissive environment for neoplastic progression. Furthermore, microbial metabolites can hijack key oncogenic signaling pathways, such as Wnt/β-catenin and PI3K/Akt, to drive uncontrolled cellular proliferation and metastasis. However, systematic reviews integrating how intratumoral microbiomes influence CRC through immune regulation, metabolic reprogramming, and oncogenic pathway activation remain scarce, which motivates this comprehensive analysis.

This review will first delineate the biological features of the intratumoral microbiome and its epidemiological association with CRC. We will then focus on three key mechanisms: (1) microbe-induced genomic mutations and epigenetic modifications; (2) microbiota-immune interactions driving chronic inflammation; and (3) intratumoral microbiome regulating oncogenic signaling pathways. Finally, we will discuss clinical translation strategies targeting the microbiome and outline current technical challenges and future directions. By synthesizing cutting-edge research across multiple dimensions, this review aims to provide a theoretical framework for microbiome-based CRC research and precision therapeutics.

## Tools and techniques for decoding the intratumoral microbiome

Unraveling the complexities of the intratumoral microbiome necessitates a sophisticated methodological arsenal, designed to overcome its defining challenge: extremely low microbial biomass amidst an overwhelming host nucleic acid background. This creates a high vulnerability to contamination, making rigorous experimental controls and advanced bioinformatic filtering the non-negotiable foundation of any reliable study. The field has evolved from mere census-taking to achieving high-resolution, functional, and spatial insights through an integrated multi-omics approach.

### Single-cell and spatial technologies for cellular context

The field has moved beyond traditional bulk sequencing approaches, striving to resolve the distribution and function of intratumoral microbiome at single-cell and spatial levels, thereby overcoming the key biological heterogeneity overlooked in earlier studies due to population averaging. Single-cell metagenomics technologies, such as INVADEseq, isolate individual host cells and detect intracellular microbes, enabling direct correlation between the colonization of specific bacteria and transcriptomic changes in host cells. This provides evidence for elucidating the specific mechanisms by which intracellular microbes influence processes such as tumor metastasis or inflammation regulation [13, 14]. However, microbial single-cell sequencing has long faced numerous technical challenges: bacterial cells are small, genetic material yield per cell is low, cell wall structures vary significantly, and traditional culture-dependent methods fail to cover uncultivable microbial taxa, making it difficult to capture rare variants or cell-to-cell heterogeneity driven by dynamic mechanisms such as phase variation [15–17]. Recently developed droplet microfluidics platforms, such as DoTA-seq, miniaturize reactions to picoliter volumes and integrate hydrogel encapsulation, single-cell lysis, targeted multiplex PCR, and molecular barcoding to achieve high-throughput, quantitative analysis of genetic features of individual microbial cells within complex communities [18]. This technology not only avoids reliance on complex microfluidic manipulation modules, enhancing accessibility and reproducibility, but also enables systematic resolution of microbial genomic variations—such as horizontal gene transfer, copy number variations, and mutation accumulation—at the single-cell level. Technological advancements have further expanded our understanding of microbial distribution patterns within tissues and their interactions with the host microenvironment. The application of spatial transcriptomics, such as 10 × Visium and NanoString CosMx, allows in situ mapping of microbial spatial distributions while preserving tissue architecture. As demonstrated by Galeano Niño et al., this technology can reveal non-random patterns of microbial localization within tumors, such as the enrichment of bacteria in specific regions characterized by low vascularization, immune richness, and concomitant local immunosuppression [19]. This spatial localization, associated with host cell states and the spatial exclusion of immune cells, highlights the potential role of microorganisms in shaping tumor heterogeneity and the immune microenvironment.

### High-resolution sequencing for strain-level discrimination

Short-read sequencing often fails to distinguish closely related microbial strains. Long-read technologies (PacBio HiFi, Oxford Nanopore) enable full-length 16S sequencing, permitting species- or strain-level identification critical for recognising pathogens such as colibactin-producing E. coli. Advanced bioinformatic tools (MetaPhlAn4, mOTUs) further enhance resolution in re-analyzing existing datasets, uncovering previously missed low-abundance taxa.

Short-read sequencing technologies, while high-throughput and cost-effective, often fail to distinguish between closely related microbial strains due to their limited read length, which prevents the capture of full-length phylogenetic markers or strain-specific genomic variants. This limitation is being overcome by long-read sequencing platforms, such as PacBio HiFi and Oxford Nanopore, which enable the sequencing of the entire ~ 1500 bp 16S rRNA gene. This full-length context provides a significantly higher phylogenetic resolution, permitting robust discrimination not only at the species level but often at the strain level, which is critical for identifying specific pathogens such as colibactin-producing E. coli [20–22]. The superiority of full-length 16S sequencing over partial variable region sequencing has been demonstrated, showing that while variable regions can identify genera, they are frequently inadequate for resolving species and strains [23]. However, the potential of this approach can be limited by platform-specific sequencing errors, such as systematic deletions in homopolymer regions in PacBio Circular Consensus Sequencing reads, which can obscure the detection of true single-nucleotide polymorphisms (SNPs) that define intragenomic variation or closely related strains [23–25]. Beyond 16S rRNA gene sequencing, the application of long-read metagenomics allows for strain-level characterization by assembling complete genomes from complex communities, thereby enabling the detection of strain-specific genes, such as those encoding virulence factors or antibiotic resistance. Advanced bioinformatic tools are crucial for leveraging these long-read data. Tools like MetaPhlAn4 and mOTUs utilize clade-specific marker genes to provide more accurate taxonomic profiles at high resolution. These tools are particularly valuable for re-analyzing existing datasets, as their improved databases and algorithms can uncover previously missed low-abundance taxa and resolve strain-level differences that were collapsed in short-read analyses [25, 26]. Furthermore, the analysis of intragenomic 16S gene copy variant SNPs—a feature uniquely accessible with high-accuracy long reads—has been shown to produce highly similar substitution profiles for closely related taxa, providing a robust method for species-level identification and even distinguishing between strains of the same species [25, 27]. As genomic databases continue to expand with more complete reference genomes derived from long-read technologies, the taxonomic precision of these classification algorithms is poised to improve substantially.

### Multi-omic integration for functional insight

To move beyond taxonomy and spatial location to functional insight, multi-omic approaches are essential. Meta-transcriptomics identifies actively transcribed microbial pathways in situ, such as virulence gene expression [28]. Metabolomics (e.g., LC–MS) detects microbial metabolites and correlates them with host phenotypic changes [28–30]. Integration of these data via machine learning can identify predictive host-microbe functional relationships and potential therapeutic targets, ultimately deciphering the functional consequences of the spatial microbial organization now being uncovered [31, 32].

### Direct visual validation and capture

Sequencing-derived hypotheses require confirmation through visualization. Multiplex fluorescence in situ hybridization [33] and D-alanine labelling [34, 35] offer specific detection and localization of live bacteria within tumor sections. Transmission electron microscopy remains the gold standard for ultrastructural confirmation of intracellular bacteria [36]. To overcome host background, microfluidics and fluorescence-activated cell sorting are adapted to physically separate microbial cells for downstream analysis, significantly improving detection sensitivity [37, 38].

## The role of the intratumoral microbiome in tumor initiation and progression

Armed with these advanced methodologies for decoding the intratumoral microbiome, the field has logically proceeded to a deeper investigation of its functional consequences. It is now evident that the intratumoral microbiome is not a mere passenger but an active participant in the multi-stage pathogenesis of cancer. This section will synthesize the compelling evidence outlining how these microbial communities contribute to both the initial transformation of cells—driving tumor initiation through genomic instability, chronic inflammation, and the activation of carcinogenic pathways—and the subsequent lethal phases of the disease—orchestrating tumor progression and metastasis by modulating oncogenic signaling, remodeling the immune microenvironment, and facilitating the formation of metastatic niches.

### Mechanisms of tumor initiation

**Fig. 1:**
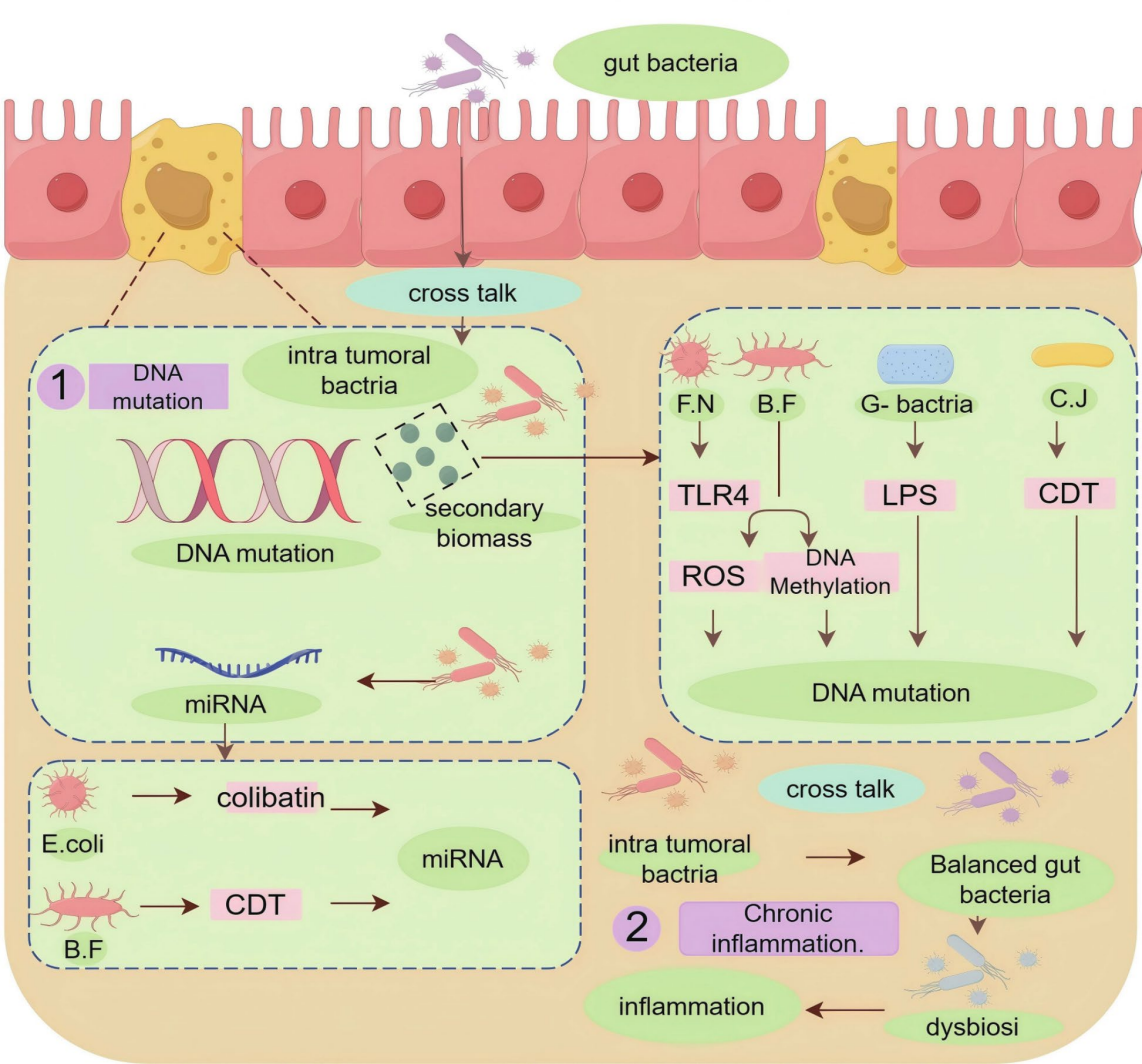
Microbial influences on tumor development within tumors. The Figure depicts the two main pathways by which microbial influences within tumors affect thedevelopment of CRC: 1) DNA mutations and 2) chronic inflammation. FN: F nucleatum. BF: Bacteroides fragilis. C.J: Campylobacter jujuni. CDT: cytolethal distending toxin

The occurrence of CRC is a complex process. Among numerous CRC patients, the etiology may involve genetic factors, environmental factors, unhealthy diet, or age-related factors, among others. [39, 40] In recent years, an increasing number of studies have indicated that intratumoral microbiome plays a significant role in the development of CRC and should be considered as an essential contributing factor. (Table 1) This article classifies the mechanisms by which intratumoral microbiome affects CRC into three main categories: inducing DNA mutations, triggering chronic inflammation, and activating oncogenic pathways (Fig. 1).

**Table 1:** Influence of Intra-tumor microbio on the occurrence of CRC

Bacteria	Secretory substance	Key results	Reference
*Campylobacter jujuni*	CDT	DNA double strand break	[41]
*Bacteroides fragilis*	BFT	Down-regulation of miR-149-3P leads to inflammation	[42]
*F. nucleatum*	genotoxin-UshA	DNA damage	[43]
*Bacteroides fragilis*	BFT, CDT	Up-regulation of spermine oxidase in intestinal epithelial cells to induce the production of ROS	[44]
*Bacteroides fragilis*	BFT	RNA methylation leads to inflammation	[45]
*F. nucleatum*		Cause kras mutation and microsatellite instability	[46]
*Campylobacter jujuni*	CDT	DNA double-strand breaks	[47]
Multiple	LPS	Activation of pattern recognition receptor NLRP3	[47–50]
Multiple		Activation of pattern recognition receptor TLR4 induces inflammation	[51–54]

*CDT* cytolethal distending toxin, *BFT* bacteroides fragilis toxin, *LPS* lipopolysaccharide, *ROS* reactive oxygen species, *NLRP3* NOD-like receptor thermal protein domain associated protein 3, *TLR4* Toll-like receptor 4

#### Induction of genomic instability

DNA mutations are one of the primary factors through which microbiota influences the onset of CRC [55, 56]. Intratumoral microbiome is characterized by high immune infiltrative properties and primarily localizes within tumor cells. The invasion of bacteria within the cells causes efficient and lethal damage to cellular DNA, often through the action of their exotoxins [57, 58]. Exotoxins, a class of toxic substances produced by bacteria and released into the surrounding environment, play a crucial role in bacterial infections and pathogenesis. They exert detrimental effects on host cells, tissues, and the immune system through diverse mechanisms, leading to cell death, tissue inflammation, and various disease-associated pathological changes [59]. Within the TME, exotoxins secreted by tumor-associated microbiota directly impact the DNA of tumor cells [58, 60].

*Campylobacter jejuni* is a common infective strain found in the intestinal tract. After invading the TME, it can produce cytolethal distending toxin (CDT), which enters target cells’ cytoplasm and induces cell cycle arrest at the G2/M phase, leading to increased DNA breakage and deformation in cells [61, 62]. Research has shown that CDT released by *Campylobacter jejuni* within the TME leads to double-stranded DNA breakage, promoting CRC [41, 61]. CDT is not exclusive to Campylobacter jejuni; many bacteria, such as *Aggregatibacter* [63] and *Escherichia coli* [64], can produce CDT and have similar effects on CRC. These bacteria have the ability to colonize within tumors, and various bacterial toxins, including Bacteroides fragilis-produced BFT and CDT, play crucial roles in the occurrence of CRC by increasing the risk of DNA mutations, downregulating cell apoptosis signals, and promoting cancer development. These toxins can induce DNA single-strand breaks, leading to the onset of CRC [65, 66].

*Fusobacterium nucleatum* is a well-studied intratumoral microbiome associated with the occurrence and metastasis of CRC. In 2024, through gene enrichment analysis of various *F. nucleatum* strains, Martha Zepeda-Rivera and colleagues identified and confirmed the significant enrichment of the Fna C2 subtype in CRC, establishing its important ecological role within this context [67]. Several metabolites secreted by *F. nucleatum*, such as tryptophan metabolites, affect cancer development through various pathways. *F. nucleatum* also secretes cytotoxins that significantly impact DNA mutations in CRC cells. Studies have shown that genotoxin-UshA, produced by *F. nucleatum*, acts on intestinal epithelial cells, inducing double-stranded DNA breaks and promoting the development of CRC [52, 68].

In addition to the direct impact of exotoxins on DNA mutations, the production of reactive oxygen species (ROS) is another important intermediary element. *Bacteroides fragilis* upregulates the expression of spermine oxidase in intestinal epithelial cells, inducing ROS production [69]. Exotoxins such as CDT and BFT can also influence ROS production, leading to CRC. Other intratumoral microbiome can affect DNA mutations through alternative pathways. For example, *F. nucleatum* can mediate KRAS mutations and microsatellite instability, resulting in DNA mutations that lead to cancer [70, 71]. And pks + *Escherichia coli* can affect the mismatch repair system of DNA, leading to double-stranded DNA breaks and influencing CRC [72].

#### Chronic inflammation

Chronic inflammation has long been recognized as a significant factor in the development of CRC [73]. It can lead to sustained damage to the colorectal mucosa and initiate an inflammatory response. These reactions promote intestinal cell proliferation, induce DNA damage, and thereby elevate CRC risk. Chronic inflammation also contributes to abnormal growth and variability of the gastrointestinal mucosa, giving rise to precursor lesions such as inflammatory polyps and inflammation-associated colorectal adenomas [74]. Both intratumoral microbiome and gut microbiota play pivotal roles in mediating chronic inflammation and its progression to CRC [75]. Specifically, this article outlines two mechanisms by which intratumoral microbiome promote chronic inflammation: (1) Through epigenetic modifications, it influences the initiation of inflammation, thereby enhancing inflammatory factor production and impacting DNA methylation, which can lead to inflammation-induced carcinogenesis. (2) Interaction between intratumoral microbiome and gut microbiota results in dysbiosis, contributing to chronic inflammation [76–78].

##### Epigenetic modifications

Pathogenic bacteria can directly interfere with the epigenetic regulation of host cells through their surface components. The surface protein FadA of *Fusobacterium nucleatum* plays a central role in this process. Its active complex, FadAc, specifically binds to the EC5 domain of host E-cadherin [79]. This binding not only mediates bacterial adhesion and invasion but also induces phosphorylation and internalization of E-cadherin, thereby stabilizing β-catenin and promoting its nuclear translocation. Within the nucleus, β-catenin activates the TCF/LEF family of transcription factors, driving the expression of proto-oncogenes such as MYC and CCND1, as well as key pro-inflammatory cytokines including interleukin (IL)−6 [80, 81]. Notably, the sustained activation of this FadA-triggered oncogenic signaling pathway is closely associated with the remodeling of the host cell's epigenetic landscape—for instance, it can induce alterations in histone modifications, such as the upregulation of activating marks like H3K27ac, thereby reinforcing a pro-inflammatory gene expression program [52, 82]. In addition, the direct binding of F. nucleatum to the RNA helicase DHX15 has been shown to remodel RNA splicing and post-transcriptional regulatory networks, driving tumor progression in KRAS p.G12D-mutant colorectal cancer [46].

Microbial metabolites serve as crucial intermediaries in epigenetic regulation, influencing the host's epigenetic landscape through diverse molecular mechanisms.

*Bacteroides fragilis* secretes the B. fragilis toxin (BFT), which alters the expression and activity of host DNA methyltransferases. This perturbation leads to genome-wide DNA hypomethylation coupled with localized promoter hypermethylation; an epigenetic state closely associated with sustained activation of inflammation-related genes [42]. Similarly, the microbiota-derived metabolite trimethylamine N-oxide (TMAO) can perturb the host epigenome by inhibiting S-adenosylhomocysteine hydrolase (SAHH) [83]. This inhibition elevates S-adenosylhomocysteine (SAH), a potent feedback inhibitor of methyltransferases, further disrupting DNA methylation patterns and potentially contributing to the silencing of tumor suppressor genes. Beyond DNA methylation, short-chain fatty acids (SCFAs), particularly butyrate produced by commensal bacteria through dietary fiber fermentation, act as potent inhibitors of class I and IIa histone deacetylases (HDACs). By enhancing acetylation at histone H3K9 and H3K27 residues, SCFAs promote the transcriptional activation of anti-inflammatory genes while suppressing pro-inflammatory cytokines, thereby fostering an anti-inflammatory epigenetic state in the gut microenvironment [84].

Interference with microRNA (miRNA) expression profiles represents another crucial dimension of microbial epigenetic influence. Specific pathogens can precisely modulate host miRNA expression either directly or via metabolite production. For example, *B. fragilis* downregulates miR-149-3p, thereby relieving its suppression of the IL-17 signaling pathway. Similarly, colibactin-producing *Escherichia coli* downregulates miR-18a and miR4802 through its genotoxic activity, exacerbating IL-17-driven inflammatory responses [42, 85]. These pathogen-induced perturbations in miRNA networks disrupt the regulation of key pro-inflammatory factors, providing an additional epigenetic explanation for how intratumoral microbiota fuels colorectal inflammation and carcinogenesis.

This multi-layered, multi-mechanistic epigenetic reprogramming enables intratumoral microbes to precisely calibrate the inflammatory tone of the tumor microenvironment. Understanding these interactions offers novel perspectives on colorectal cancer pathogenesis and opens avenues for the development of epigenetically targeted therapeutic strategies. Future studies should focus on delineating the precise molecular dialogue between specific bacterial strains and their epigenetic targets, as well as the dynamic evolution of these interactions across different stages of tumor progression.

##### Crosstalk with gut microbiota

In CRC, the intratumoral microbiome and the gut microbiota are closely linked, often collaborating in carcinogenesis. A key similarity lies in their shared capacity to influence CRC initiation and progression through metabolites that modulate immune responses and oncogenic pathways [86]. However, Despite its low biomass, the intratumoral microbiome interacts intimately and potently with the local tumor immune microenvironment. [87]. The gut serves as a vast reservoir of beneficial microbes, including Bifidobacterium, Bacteroides, Lactobacillus, and Prevotella, which are crucial for maintaining gut homeostasis. Many of these, such as Enterococcus, Streptococcus, Bifidobacterium, Lactobacillus, Prevotella, and Actinomyces, produce short-chain fatty acids (SCFAs) that can directly inhibit tumor growth and modulate B cell and macrophage responses to suppress CRC [88]. Furthermore, bile acid-metabolizing bacteria such as Bacteroides, Lissabonella, and Clostridium scindens have been shown to impact interferon-gamma (IFN-γ) production and natural killer (NK) cell activity, thereby contributing to CRC prevention [89, 90]. The intratumoral microbiome can disrupt this homeostatic balance in two primary ways. First, it directly influences gut microbial composition. Studies indicate that CRC patients exhibit an enrichment of bacteria such as B. fragilis, *F. nucleatum*, Saccharopolyspora, Pseudoxanthomonas, and Streptomyces within tumors, while their gut microbiota shows a concurrent reduction in beneficial genera such as Enterococcus, Lactobacillus, and Prevotella [91]. Second, intratumoral bacteria can interfere with the antitumor effects of gut microbiota within the TME. As integral components of the TME, pathobionts such as Fusobacterium spp., Bacteroides fragilis, and Streptococcus gallolyticus often exert opposing effects on immune inflammatory factors and immune cells compared to the beneficial gut species [92].

Conversely, the gut microbiota significantly influences the intratumoral niche. The partial taxonomic overlap between the two communities supports the theory that the gut is a key source of intratumoral microbes. This functional crosstalk is demonstrated by studies showing that FMT can alter the composition of the intratumoral microbiome and affect survival in mouse models [92, 93].

#### Activation of carcinogenic pathways

The onset of CRC results from a complex interplay of various factors, with the activation of carcinogenic pathways being pivotal to its development. Several classical pathways associated with CRC are widely acknowledged. Among these, the Wnt/β-catenin signaling pathway stands out as a prominent carcinogenic pathway, crucial for tumorigenesis and tumor progression. Activation of this pathway stimulates cell proliferation and differentiation, culminating in tumor formation [94].The PI3K/Akt signaling pathway, extensively implicated in numerous tumors, plays roles in essential biological processes including cell proliferation, survival, and metastasis. Its heightened activity is closely linked to the onset and progression of several cancer types. In CRC, activation of the PI3K/Akt signaling pathway significantly contributes to tumor development [95].

Intratumoral microbiome play a critical role in this process, primarily through the modulation of microbial pattern recognition receptors (PRRs) [96, 97]. PRRs, such as Toll-like receptors (TLRs), Nucleotide-Binding Oligomerization Domain-Like Receptors (NLRs), Retinoic Acid-Inducible Gene I-Like Receptors (RLRs), and C-Type Lectin Receptors (CLRs), are protein molecules present on animal and plant cell surfaces. They function as crucial components of the immune system, detecting specific molecular patterns exhibited by bacteria and other microorganisms to initiate immune responses, aiding the host in defending against pathogenic infections [98]. TLRs and NLRs, particularly abundant on cell membranes, cytoplasm, and endoplasmic reticula, are responsible for recognizing intratumoral microbiome.

Among the PRRs studied in relation to CRC, TLR4 and NLRP3 have received extensive attention. TLR4, a significant pattern recognition receptor, influences CRC development through its recognition of lipopolysaccharides (LPS) from intratumoral microbiome [99–101] Microorganisms within tumors, such as *F. nucleatum*, Bacteroides fragilis, and *Campylobacter jujuni*, activate TLR4 via LPS secretion. This activation, mediated by MYD88, triggers the classical AKT and NF-kB pathways, ultimately initiating CRC [98]. NLRP3, also known as Nucleotide-Binding Oligomerization Domain-Like Receptor Family Pyrin Domain-Containing 3, is a critical member of the NLR family, primarily localized in the cytoplasm. It functions in detecting and responding to microbial, cellular stress, and damage signals [102, 103]. Studies indicate that NLRP3 plays a significant role in the CRC TME. Its activation correlates with increased inflammation and likely contributes to tumor development. Once activated, NLRP3 forms an inflammasome complex, triggering inflammatory responses and cellular apoptosis. Activation of NLRP3 in the TME is typically induced by various pathogen-associated molecular patterns (PAMPs), such as E. coli, Bacteroides fragilis, and other bacterial strains [50]. Escherichia coli plays a significant role in the development of CRC liver metastasis (CRLM). This bacterium induces the lactylation of retinoic acid-induced gene 1 (RIG-I), which subsequently inhibits the nuclear factor-κB (NF-κB) signaling pathway. This inhibition promotes lactate production in M2 macrophages. Furthermore, the lactylation of RIG-I impedes the recruitment of NF-κB to the Nlrp3 promoter in macrophages, resulting in a decreased transcriptional activity. These findings elucidate the potential mechanisms by which E. coli influences tumor metabolism and immune response in the context of CRLM [104]. Furthermore, NLRP3 detects specific microbial factors within tumors, including LPS from intratumoral microbiome and beta-glucan from invading fungi. Upon activation, the NLRP3 inflammasome influences pathways involved in cell proliferation, PI3K/Akt, and MAPK, and affects ROS production, contributing to CRC development by impacting DNA damage [105–107].

### Mechanisms of tumor progression and metastasis

#### Modulating oncogenic signaling pathways

**Fig. 2:**
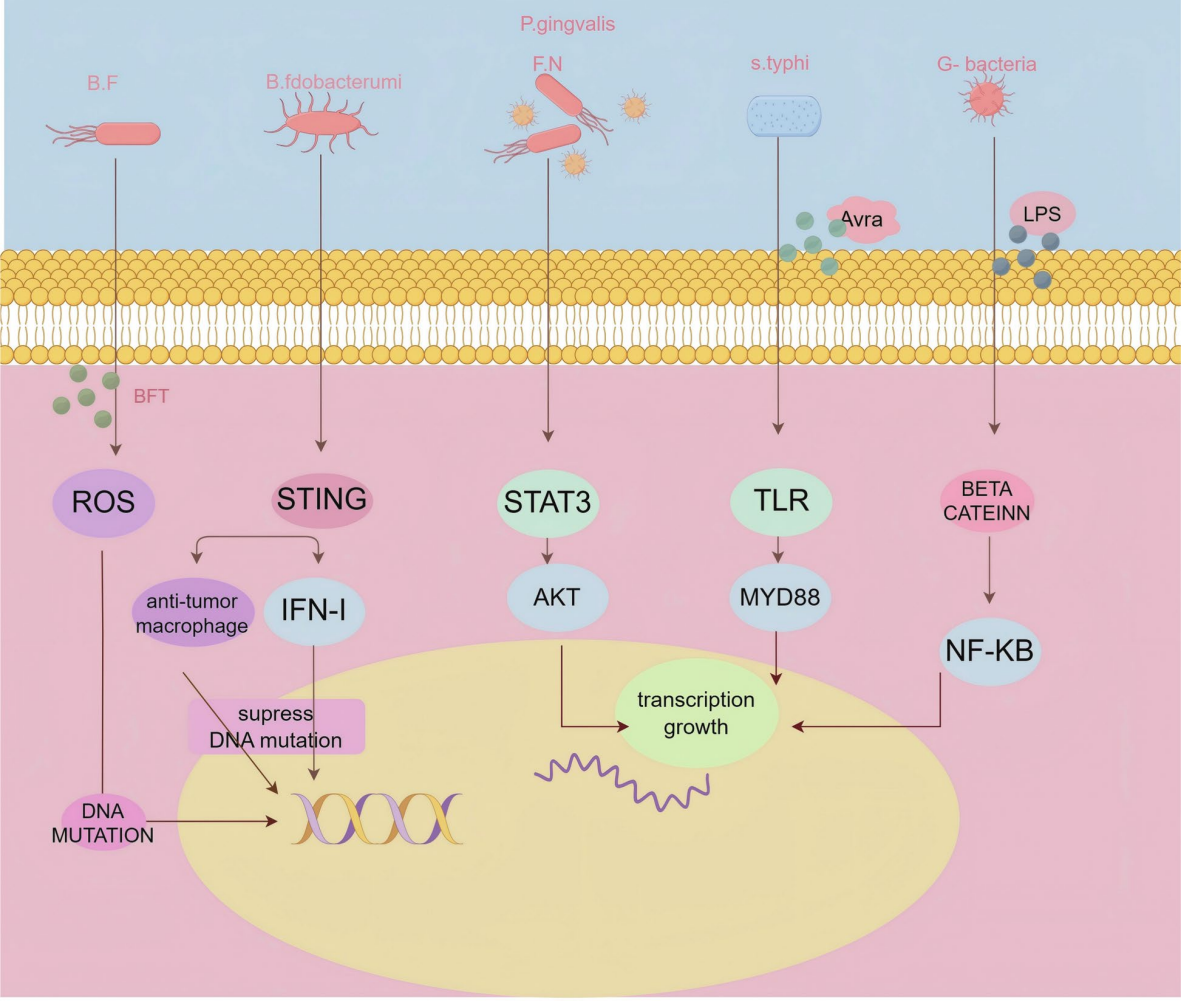
Microbial within tumors affects CRC by influencing related pathways. The Figure depicts the five pathways by which microbial influences within tumors affect CRC: 1) ROS, 2) STING, 3) STAT3, 4) TLR, and 5) B-catenin. The different colors ofsolid lines represent different forms of pathway implementation. F.N: F nucleatum. B.F: Bacteroides fragilis

Recent years have seen extensive research on the influence of tumor-associated microbes on various pathways. In this study, we summarize the findings on pathways affected by these microbes in CRC, including ROS, β-catenin, NF-κB, STING, PI3K, and others, which will be discussed in detail (Fig. 2) (Table 2).

**Table 2:** Pathways that affect the development of CRC

Signing pathway	Cancer promoting/anti-cancer	bacteria	Mechanism	*Reference*
ROS	Cancer promoting	Bacteroides fragilis	Up-regulation of spermine oxidase in intestinal epithelial cells to induce the production of ROS	[108]
STAT3	Cancer promoting	P.gingivalis Bacteroides fragilis,F.nucleatum	Bacterial exotoxins and metabolites activate immune response and activate classical STAT3 to dredge collaterals	[109–111]
TIGIT	anti-cancer	Bifidobacterium A. muciniphila Lactobacillus murine,Bacteroides uniformis	Production of Fap2 protein activates tigit to dredge collaterals, affecting macrophage M1 polarization and T cell activation	[112]
TLR4/MYD88	Cancer promoting	mutiple	Bacterial surface antigen activation pattern recognition receptor activates TLR/MYD88 to open collaterals	[95]
beta catenin	Cancer promoting	Ecoli,Bacteroides fragilis,F. nucleatum	The expressed adhesion molecule FadA binds to e-cadherin on host cells	[108]
NLRP3	Cancer promoting	Multiple	Bacterial surface LPS activation pattern recognition receptor activates TLR/MYD88 to dredge collaterals	

*ROS* reactive oxygen species, *STAT3* signal transducer and activator of transcription 3, *TIGIT* T cell immunoreceptor with Ig and ITIM domains, *TLR* toll-like receptor 4, *MYD88* myeloid differentiation primary response 88, *NLRP3* NOD-like receptor thermal protein domain associated protein 3, *LPS* lipopolysaccharide

##### ROS signaling pathway

ROS refer to the oxidative-reductive reactions of enzymes such as adenosine nucleotide phosphatase or those involved in mitochondrial respiration. The roles of ROS in cancer are diverse, including DNA damage, promotion of cell proliferation, evasion of apoptosis, induction of tissue invasion, and angiogenesis [113, 114]. ROS also play a significant role in one of the most recognized metastasis mechanisms, epithelial-mesenchymal transition (EMT) [115]. It has been reported that the toxin produced by BFT increases ROS levels in intestinal epithelial cells, leading to DNA oxidation and damage, resulting in malignant cell transformation [44]. P. aeruginosa can produce oxidative stress, leading to gene mutations, particularly gyrA gene mutations, which result in acquired fluoroquinolone resistance [116]. Apart from epithelial cells, macrophages, neutrophils, and fibroblasts in the TME can also produce ROS. BFT produced by Bacteroides fragilis can promote high ROS levels in myeloid cells. DNA damage induced by mitochondrial ROS decreases NAD + levels, resulting in the senescence of M1-like macrophages [117]. ROS produced by tumor-associated macrophages (TAMs) trigger the activation of matrix metalloproteinases, leading to EMT in adjacent epithelial cells and increased tumor cell invasion [118]. Furthermore, myeloid-derived suppressor cells (MDSCs) suppress T cell function by generating peroxynitrite in a ROS-dependent manner [119].

##### Signal transducer and activator of transcription 3 (STAT3)

STAT3 is a signaling and transcription activation protein and a member of the STAT protein family. STAT3 plays a crucial role in multiple biological processes, including embryonic development [120], cell growth [121], differentiation [122], and immune regulation [123]. Microbes such as *Bacteroides*, *Enterococcus, and Bifidobacterium* can influence the abnormal activation of STAT3 through downstream signaling pathways generated by their pattern recognition receptors, leading to the occurrence and development of CRC [109–111]. *F. nucleatum* can also affect the expression of interleukin 22, thereby influencing the downstream expression of STAT3, and consequently impacting the occurrence and development of CRC [124].

##### β-catenin signaling

Numerous microorganisms have been identified as activators of the β-catenin signaling pathway, including *S. typhi*, *F. nucleatum*, and *B. fragilis*, among others [125, 126]. The activation of the β-catenin signaling pathway is often closely related to the secretion of E-cadherin. Notably, *Salmonella*’s effector protein AvrA can increase the expression of Wnt, Wnt receptor Frizzled 7, and T cell factor/lymphoid enhancer factor-1. Additionally, AvrA can regulate various post-transcriptional modifications of β-catenin, collectively regulating the activity of β-catenin [127, 128].

##### STING signaling pathway

STING, a cytoplasmic protein involved in DNA sensing, can be activated to induce the expression of IFN-β and other pro-inflammatory genes upon binding to cyclic dinucleotides, such as cyclic di-guanosine monophosphate, cyclic di-adenosine monophosphate, and 2′3’-cyclic GMP-AMP (cGAMP) [129]. During pathogen infection, cyclic GMP-AMP synthase (cGAS) can directly sense pathogen-derived DNA, catalyzing the synthesis of cGAMP from ATP and GTP, which subsequently activates the downstream host cell STING/tank-binding kinase 1/interferon regulatory factor 3/IFN-β signaling pathway [130, 131]. The expression of STING is observed in macrophages, dendritic cells, lymphocytes, endothelial cells, and epithelial cells. Extensive research has demonstrated the robustness of the cGAS/STING signaling pathway in eliciting a potent immune response [132].

The regulation of macrophage polarization towards the M1 phenotype and the manifestation of potent anti-tumor effects have been observed. In various tumor models, bacteria such as Fusobacterium nucleatum can generate STING agonists, which target monocytes and drive polarization towards anti-tumor macrophages [133]. This process facilitates the activation of natural killer (NK) cells and their interaction with dendritic cells through the production of IFN-I. Moreover, Bacteroides fragilis can directly activate the STING signal in dendritic cells, leading to their activation. However, the mode of STING activation, whether through the recognition of bacterial DNA by cGAS or other bacterial products, remains uncertain. Within macrophages, the IFN-I produced by the STING signaling pathway can activate tumor-infiltrating Batf3(Basic Leucine Zipper Activator 3) dendritic cells and tumor antigen-specific CD8^+^ T cells [134]. In studies related to gut microbiota, the oral administration of *Lactobacillus rhamnosus GG*, combined with immune checkpoint blockade, has been shown to promote the enrichment of *Lactobacillus murinus* and *Bacteroides uniformis* in the microbiota, triggering cGAS/STING-dependent IFN-I production, dendritic cell activation, and CD8^+^ T cell recruitment in tumors [132].

#### Remodeling the tumor immune microenvironment

**Fig. 3:**
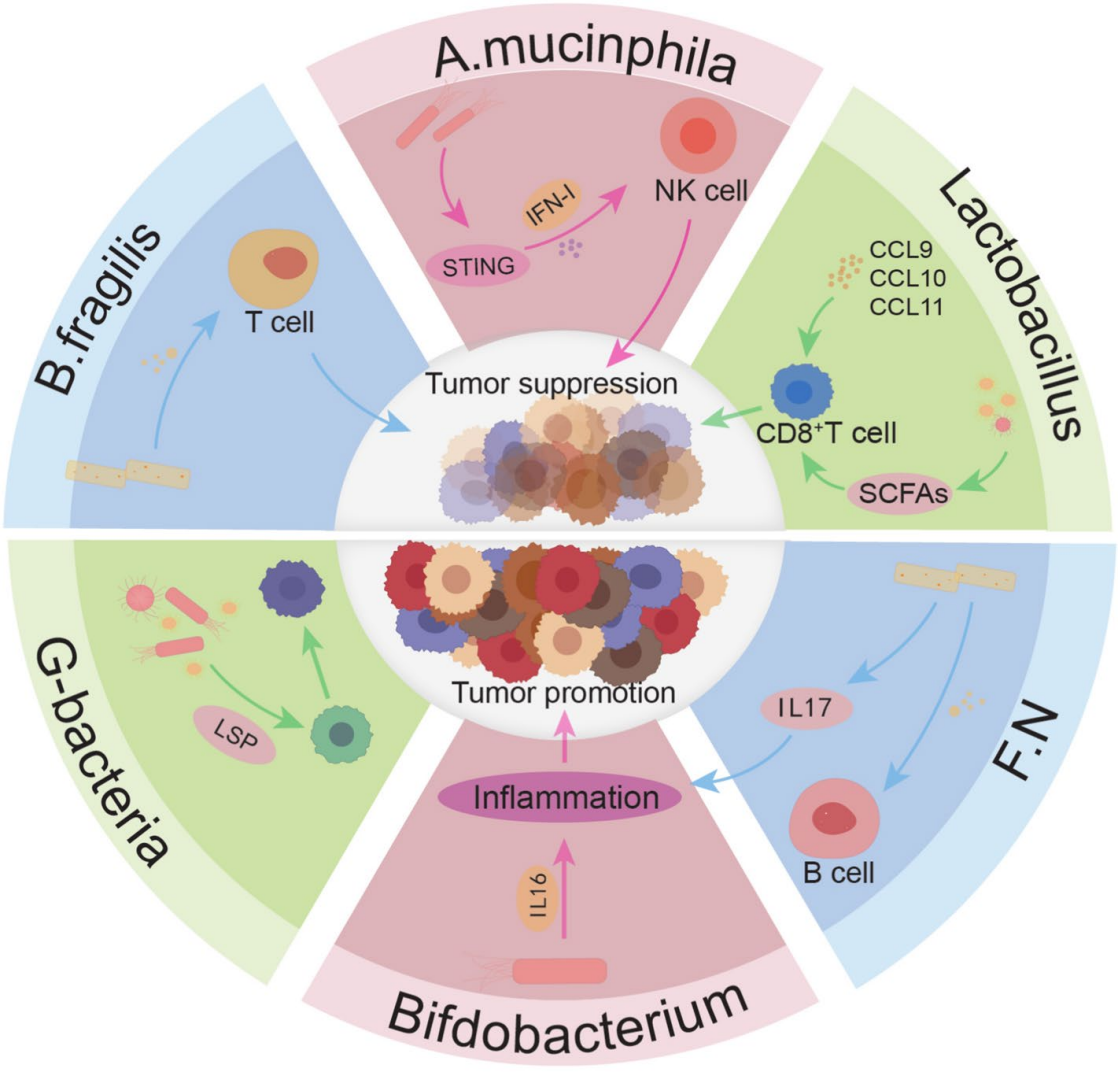
The microbiota residing within tumors can modulate the immunemicroenvironment, thereby influencing tumor development. This figure illustrateshow microbial components affect various immune cells including T cells, NKcells, B cells, and macrophages as well as inflammatory factors such as CCL, IL2. and IL-18. These interactions collectively determine whether the immuneresponse promotes or inhibits tumor progression. F.N: F. nucleatum. CCL: C–C Motif Chemokine Ligands

The intratumoral microbiome is a crucial component of the immune microenvironment, significantly impacting immune cells and immune-related inflammatory factors. This article categorizes the effects of intratumoral microbiome on CRC into two main types: direct effects on immune cells and the promotion of immune-related factors (Fig. 3) (Table 3).

**Table 3:** Intra-tumor bacteria affect immune microenvironment

Bacteria	Cancer promoting/anti-cancer	Mechanism	reference
*Lachnoclostridium*	Anti-cancer	Induction of CCLs production	[135]
*Lachnoclostridium*	Anti-cancer	induce the production of CXCL9, CXCL10 and CCL5	[136]
*Cutibacterium acnes*	Anti-cancer	enhance the infiltration of treg cells	[137, 138]
*Lactobacillus*	Anti-cancer	Increase of CD8 positive T cells through SCFAs	[139]
Multiple	Cancer promoting	Induction of IL-16	[140, 141]
*Bifidobacterium* and *Lactobacillus*	Anti-cancer	inhibit LPS-mediated IL-6 production	[142]
Multiple	Cancer promoting	Secreted IL-17 can promote B cell infiltration in tumor	[143]
,*B. fragilis F. nucleatum*	Cancer promoting	IL-2 level decreased	[144]

*CCLs* C–C motif chemokine ligands, *CXCL* C-X-C motif chemokine ligand, *SCFAs* short-chain fatty acids, *IL* Interleukin, *LPS* lipopolysaccharide

##### Regulation of T Cells and NK Cells

The intratumoral microbiome also shapes the anti-tumor immune response by promoting the activation of T cells and NK cells. For instance, bacteria such as *Mucinivorans*, *Pseudomonas*, and *Streptomyces* found in pancreatic cancer tissues promote the recruitment and activation of CD8^+^ T cells, thereby enhancing the anti-tumor immune response [145]. Patients with long-term survival exhibit higher densities of CD8^+^ T cells and granzyme B + B cells compared to short-term survival patients [146], with no significant differences observed in macrophages, regulatory T cells (Tregs), and MDSCs. Furthermore, the density of CD8^+^ T cells and granzyme B^+^ B cells is closely associated with tumor tissue and overall survival rates in pancreatic cancer patients [147, 148].

##### B cell activation and macrophage polarization

The antigens present on the surface molecules of intratumoral microbiome can be recognized by the antibody receptors on B cell membranes. This recognition process initiates the activation of B cells, leading to their differentiation and proliferation. Consequently, antibodies are produced to counteract the invasion of microbes [149, 150]. On the other hand, macrophages possess recognition receptors such as TLRs and NLRs, which detect specific molecular features of microbes known as PAMPs. The binding of these receptors to microbial-associated molecules triggers macrophage activation and induces their polarization. For instance, TLR2 and TLR4 can detect bacterial LPS and other microbial molecules, promoting macrophage polarization towards the pro-inflammatory (M1) type. Additionally, certain microbial molecules can directly or indirectly activate macrophages, inducing their polarization. Notably, extracellular secretory products from specific bacteria, such as lipopeptides and polysaccharides, stimulate macrophages to produce pro-inflammatory cytokines, further promoting the formation of M1 macrophages [151, 152].

##### Regulation of chemokines

C–C Motif Chemokine Ligands (CCLs) are a type of chemotactic factor that directs immune cells to sites of infection, inflammation, and tumors. Chemokines such as CCL2, CCL5, and CCL17 promote the migration and aggregation of immune cells such as monocytes, lymphocytes, and dendritic cells to these sites. Many intratumoral microbiome impact CCLs [135, 153]. For example, Lachnoclostridium can suppress the production of CXCL9, CXCL10, and CCL5, thereby reducing inflammation and inhibiting CRC [154]. Bile salt hydrolase (BSH) in *Bacteroides* can enhance the expression of CCL28 regulated by β-catenin in the context of colon tumors. Activation of the β-catenin/CCL28 axis leads to an increase in immunosuppressive CD25^+^ FOXP3^+^ T regulatory (Treg) cells within the TME. The blockade of the β-catenin/CCL28 axis releases immunosuppression and promotes anti-tumor responses within the tumor [155]. *Peptostreptococcus* exacerbates CRC by mediating resistance to anti-PD-1 therapy and through MDSCs. This bacterium induces the secretion of CXCL1 and recruits CXCR2^+^ MDSCs into the tumor by activating the integrin α2β1-NF-κB signaling pathway in CRC cells [156].

##### Interleukin networks

Interleukins are a type of cytokine involved in regulating the functions of the immune system. They transmit signals between immune cells and affect cell proliferation, differentiation, activation, and regulation of immune responses [157]. The impact of intratumoral microbiome on interleukins is diverse, with various mechanisms at play. Beneficial bacteria such as *Bifidobacterium* and *Lactobacillus* can produce interleukin-6 (IL-6) through LPS-dependent metabolic byproducts. Bacteria such as *Bacteroides fragilis* and *Fusobacterium nucleatum* can also alter interleukins through bacterial exotoxins. For example, Bacteroides fragilis-produced BFT can reduce the production of IL-2, promoting tumor development [144].

#### Formation of the pro-metastatic niche

The intricate crosstalk between oncogenic signaling pathways and an immunosuppressive microenvironment, as detailed in the preceding sections, does not merely fuel primary tumor growth but also critically orchestrates the multi-step process of metastasis. The culmination of these microbial-driven activities is the formation of a pro-metastatic niche—a supportive microenvironment at distant organs that primes the soil for the seeding and colonization of circulating tumor cells. The lethality of CRC primarily depends on its metastatic potential. While the 5-year survival rate for patients with localized, early-stage CRC can exceed 90%, it plummets to below 10% for those diagnosed with advanced metastatic disease [158]. The intratumoral microbiome plays a crucial role in this process. Therefore, gaining a deep understanding of the mechanisms underlying metastasis is of fundamental importance for improving the prognosis of cancer patients.

A substantial body of evidence indicates that specific intratumoral bacteria can systematically promote the metastatic cascade of CRC by modulating both the intrinsic properties of cancer cells and the TME. During the local invasion stage, *Fusobacterium nucleatum* contributes through its surface adhesin FadA, which binds to E-cadherin on CRC cells, activating β-catenin signaling. This not only promotes proliferation but also upregulates the expression of pro-inflammatory cytokines such as IL-6 and IL-8, as well as oncogenes such as MYC, thereby enhancing the invasive potential of tumor cells [53, 159]. Furthermore, enterotoxigenic *Bacteroides fragilis* secretes the BFT, a metalloprotease that cleaves E-cadherin, disrupting epithelial integrity and triggering a potent, IL-17-driven pro-carcinogenic inflammatory response [160, 161]. This sustained inflammation, characterized by the influx of myeloid cells and the release of matrix-degrading enzymes, remodels the tumor stroma and facilitates cancer cell intravasation into the circulation. Upon entering the circulatory system, intratumoral bacteria may facilitate "co-migration" by forming aggregates with circulating tumor cells. Compelling evidence for this direct role in metastasis comes from the detection of viable *Fusobacterium nucleatumin* CRC liver metastases, with bacterial strains matching those in the primary tumor, suggesting a protective role for bacteria during hematogenous dissemination. Mechanistically, intratumoral bacteria can enhance tumor cell survival in the bloodstream by reinforcing the actin cytoskeleton, helping cells resist fluid shear stress [162]. Additionally, bacteria such as *Escherichia colican* promote metastasis by disrupting the gut-vascular barrier and disseminating to the liver to form a pre-metastatic niche, thereby recruiting and facilitating the colonization of CRC cells in the liver [152]. During the colonization stage, intratumoral bacteria further support the establishment of metastases by modulating the local immune microenvironment. For instance, tumor regions enriched with *Fusobacterium nucleatum* often exhibit reduced infiltration of CD4^+^ and CD8^+^ T cells, indicating suppression of anti-tumor immunity [163]. Similarly, studies in breast cancer models have shown that intratumoral bacteria can dampen T cell activity by modulating the β-catenin signaling pathway, thereby enhancing immune evasion [164]. Moreover, antimicrobial treatment in a murine melanoma model significantly reduced lung metastasis, concomitant with decreased regulatory T cell activity and enhanced activation of T cells and NK cells, further confirming the role of the microbiota in shaping a pro-metastatic niche [165]. Although these studies illuminate the multifaceted roles of the intratumoral microbiome in CRC metastasis, key questions remain regarding their precise origins, the mechanisms controlling their abundance, and their systemic impact on the TME. Future research must focus on elucidating the specific molecular pathways through which intratumoral microbiome interact with the immune system, thereby identifying novel therapeutic targets for intercepting CRC metastasis

## Diagnostic and prognostic biomarker potential

Research on the intratumoral microbiome in CRC is uncovering intricate links between microbial communities and carcinogenesis with exceptional resolution. These insights not only advance our understanding of CRC pathogenesis but also highlight the intratumoral microbiome as a promising target for clinical translation, holding considerable potential as a source of diagnostic and prognostic biomarkers.

The diagnostic utility of the intratumoral microbiome is underscored by distinct compositional shifts in CRC tissues. Compared with healthy tissues or controls, CRC tumors exhibit characteristic microbial signatures, consistently marked by an increased abundance of taxa such as *Fusobacterium*, *Leptotrichia*, *Campylobacter*, and oral pathobionts including *Fusobacterium nucleatum *[5, 166, 167]. These differentially abundant microbes offer a molecular basis for discriminating tumor from normal tissue and potentially identifying CRC patients among healthy individuals. Nevertheless, developing robust diagnostic models requires accounting for the pronounced spatial heterogeneity of the intratumoral microbiome. This heterogeneity is multidimensional: first, microbial composition changes dynamically throughout the colorectal adenoma–carcinoma sequence; second, significant variations can occur between different sampling sites within the same tumor [12, 168]. Importantly, tumor anatomical location—proximal versus distal—also strongly influences microbiota composition. For example, *Prevotella* and *Firmicutes* are more enriched in proximal tumors, whereas *Bacteroidetes* and certain pathogens are more characteristic of distal tumors [5]. These observations emphasize that spatial origin and tissue context must be incorporated as key variables in the development and validation of diagnostic tools based on the intratumoral microbiome. The integration of artificial intelligence (AI) and machine learning is particularly promising for this task. These models can computationally deconvolute spatial heterogeneity, integrate multi-omics data, and identify robust, generalizable microbial signatures that remain predictive across diverse patient cohorts and tumor locations.

In prognosis, specific microorganisms and community-level patterns show compelling associations with patient survival. Single-Species Associations: *Fusobacterium nucleatum* is a well-validated indicator of poor prognosis. Its enrichment in tumor tissue correlates consistently with adverse clinical outcomesand is most pronounced in advanced-stage (III/IV) CRC [169]. Additionally, *Bifid obacterium* has been linked to specific pathological subtypes, such as signet-ring cell carcinoma, implying a role in shaping tumor phenotype [170]. Furthermore, the collective composition of the intratumoral microbiome—its community subtypes—serves as a powerful prognostic indicator. Mourandov et al. [171] classified the CRC intratumoral microbiome into three oncogenic community subtypes (OCS): OCS1 (dominated by *Fusobacterium*) and OCS3 (dominated by *Escherichia coli, Pseudomonas*, and *Shigella*) were associated with significantly worse overall survival, whereas OCS2 (enriched in *Firmicutes* and *Bacteroidetes*) correlated with more favorable outcomes [171]. This suggests that communities dominated by pathobionts are particularly strong predictors of poor prognosis. The functional impact of a given bacterium is not absolute but is modulated by its ecological context. Alexander et al. [172]. illustrated this complexity by identifying 13 microbial clusters in CRC. Notably, Cluster 1—enriched in the typically beneficial *Faecalibacterium prausnitzii*—was an independent predictor of poorer disease-free survival. Conversely, in Cluster 7, high abundance of a group that included the pathobionts *F. nucleatum* and *Granulicatella adiacens* predicted improved disease-free survival [172]. This finding underscores how interbacterial interactions can profoundly alter functional outputs and cautions against interpreting the biomarker significance of individual taxa in isolation. The discovery of such complex, non-linear relationships between microbial communities and clinical outcomes is greatly facilitated by unsupervised AI methods such as clustering and network analysis. Future prognostic models will likely rely on supervised deep learning to dynamically integrate these community-level patterns with host factors for more accurate survival prediction. Next-generation sequencing technologies, particularly 16S rRNA gene sequencing, have been instrumental in mapping the complex landscape of the intratumoral microbiome. Building on these tools, integrating microbial signatures—derived from tumor or stool samples—with clinicopathological data (e.g., TNM stage) to construct predictive models represents a feasible translational strategy. Researchers from the University of Melbourne and elsewhere have advanced this concept by successfully classifying CRC into three subtypes with distinct clinical, molecular, and prognostic characteristics based on intratumoral microbiome profiles [171]. This work provides a proof-of-concept for microbiota-based molecular subtyping of CRC, which may eventually guide more personalized therapeutic development and clinical management.

In summary, the intratumural microbiota constitutes an emerging and rich repository of biomarkers with broad prospects for improving the precision of CRC diagnosis, risk stratification, and prognosis. Future efforts should prioritize standardizing sampling methodologies, addressing heterogeneity-related challenges, and elucidating the functional mechanisms of microbial influence to accelerate clinical translation. Furthermore, the integration of AI and machine learning represents a transformative frontier in this field. AI algorithms are exceptionally suited to decipher the complex, high-dimensional datasets generated by microbiome sequencing. They can integrate microbial abundances, spatial distribution, community subtypes, and host clinicopathological data to build superior diagnostic and prognostic models that overcome the challenge of tumor heterogeneity. Looking ahead, AI-driven analysis will be crucial for predicting patient-specific responses to conventional and microbiome-targeted therapies, ultimately guiding the development of personalized intervention strategies and engineered microbial therapeutics.

## Therapeutic targeting of the intratumoral microbiome

This section on therapeutic applications moves from a molecular understanding of the intratumoral microbiome to its therapeutic targeting. We review strategies designed to manipulate this niche, from modulating its native function to enhance treatment efficacy, to engineering synthetic microbes and leveraging their synergy with immunotherapy.

### Modulating the microbiome to enhance conventional therapy

The efficacy of conventional and emerging therapies in CRC—including chemotherapy, radiotherapy, and immunotherapy—is significantly modulated by the intratumoral microbiome. This microbial influence is complex and dualistic, capable of either promoting treatment resistance or enhancing therapeutic response. In chemotherapy, specific intratumoral bacteria have been directly implicated in drug resistance through diverse mechanisms [173]. *Fusobacterium nucleatum* can activate autophagy pathways to confer resistance [51, 174, 175], while *Gammaproteobacteria*, including certain *E. coli* strains, can enzymatically inactivate key drugs such as gemcitabine and 5-fluorouracil (5-FU) [176–178]. Recent research has revealed that *Lactobacillus iners*, a lactate-producing bacterium, can induce resistance to both radiotherapy and chemotherapy through metabolic reprogramming of cancer cells toward increased lactate utilization [179]. Conversely, some commensal bacteria have been shown to potentiate the efficacy of oxaliplatin by modulating ROS [178]. The interaction with immunotherapy is particularly nuanced. While *F. nucleatum* is generally associated with an immunosuppressive microenvironment and resistance to immune checkpoint inhibitors (ICIs) in microsatellite-stable (MSS) tumors, it appears to sensitize microsatellite-instability-high (MSI-H) tumors to anti-PD-L1 therapy by stimulating innate immune signaling and CD8^+^ T cell infiltration [112, 133, 180]. Other bacteria, such as B*ifidobacterium adolescentis* and *Helicobacter hepaticus*, can enhance anti-tumor immunity by activating specific macrophage subsets or promoting the formation of tertiary lymphoid structures, thereby improving responses to immunotherapy [150, 181]. Collectively, these findings underscore the critical role of the intratumoral microbiome as a dynamic modulator of treatment outcomes in CRC. A deeper understanding of these host-microbe-therapy interactions is essential for developing predictive biomarkers and designing novel combinatorial strategies that target the microbiome to overcome resistance and improve patient survival.

### Strategic interventions: from depletion to engineering

**Fig. 4:**
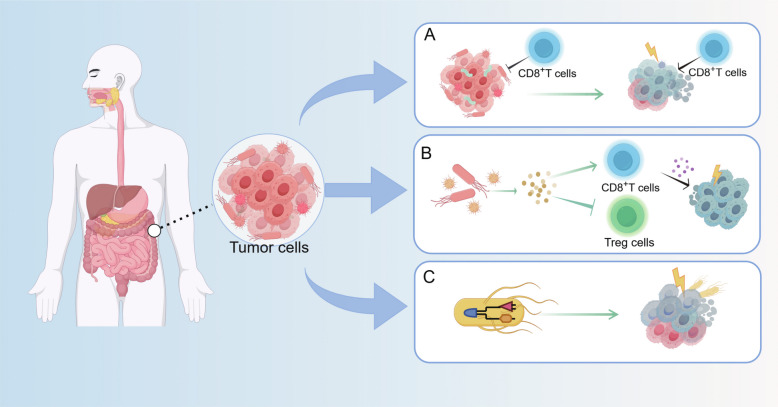
Therapeutic strategies targeting the intratumoral microbiome. **A**Targeted eradication of intratumoral bacteria. **B** Modulation of the tumor immune microenvironment via intratumoral microbiota. **C** Application of engineered microbial robots for sophisticated anti-tumor interventions

Building on the understanding of microbial influence, therapeutic strategies are evolving along three complementary fronts: precise eradication of harmful bacteria, utilization of beneficial species, and engineering of sophisticated microbial robots (Fig. 4).

#### Targeting intratumoral bacterial clearance

Although targeted eradication of tumor-associated bacteria shows considerable potential, current intervention strategies face significant challenges. Particularly noteworthy is the demonstrated interference of broad-spectrum and systemically administered antibiotics with treatment efficacy in both preclinical models and patients. In murine models of CRC and melanoma, the co-administration of broad-spectrum antibiotics with anti-IL-10/CpG oligodeoxynucleotide immunotherapy was found to compromise therapeutic outcomes by depleting the gut microbiota and reducing pro-inflammatory cytokine production. Clinical observations further confirm that systemic antibiotic use may suppress gut microbial communities, weaken anti-tumor immune responses, and diminish the effectiveness of ICIs. These findings underscore the urgent need for more precise strategies to achieve selective microbial intervention.

A paradigm for such a targeted approach is exemplified by a liposome-encapsulated antibiotic complex (LipoAgTNZ) [182], engineered to selectively eradicate tumour-associated bacteria—such as *Fusobacterium nucleatum*—within primary and metastatic murine CRC lesions without inducing gut dysbiosis. Employing this strategy, targeted bacterial clearance generates microbial neoantigens that, in turn, elicit a potent, tumour-specific CD8^+^ T cell response. This approach achieved a remarkable long-term survival rate exceeding 70% in *F. nucleatum*-infected models, effectively remodelling the immunosuppressive tumour microenvironment and stimulating systemic immunity against both infected and uninfected tumour cells. Similarly, Recent studies demonstrate that a liposome-encapsulated silver ion drug system selectively eradicated *Fusobacterium nucleatum* colonization associated with colon cancer, thereby inhibiting cancer progression [183] and a *Bacteroides fragilis*-targeting phage, VA7, selectively suppresses this bacterium and restores chemosensitivity in preclinical CRC mouse models [184]. Collectively, these findings illuminate a promising frontier in which the pharmacological targeting of the intratumoral microbiome may form the foundation for a novel class of cancer therapies.

#### Harnessing intrinsic antitumor properties

Compared to normal tissues, tumor tissues exhibit a distinct microbial community structure, and this differential microbial colonization is emerging as a critical factor influencing cancer progression. Notably, studies demonstrate that isolated intratumoral bacteria possess inherent biocompatibility and exhibit potent immunogenic antitumor properties, enabling them to preferentially colonize and proliferate within the TME while effectively eliciting potent antitumor responses [20]. Accumulating evidence indicates that within the CRC microenvironment, such beneficial microbial populations can exert antitumor effects through multiple mechanisms, including direct bacterial oncolysis, activation of host pathways via microbial metabolic interactions, and immune regulation through bacterial metabolites. These interconnected pathways converge to form a sophisticated antitumor regulatory network. Certain members of the intratumoral microbiome demonstrate the ability to specifically colonize tumor tissues and directly induce tumor cell lysis. These bacteria exploit the unique TME for selective proliferation, secreting toxin proteins or enzymes that disrupt tumor cell membrane integrity and induce cell death. Of particular interest is the AUN bacterial consortium [185], composed of *Proteus mirabilis* and *Rhodopseudomonas* palustris in a precise ratio, which has demonstrated significant antitumor efficacy across multiple mouse tumor models. Recent research reveals that the AUN consortium can achieve tumor-specific proliferation and induce tumor lysis through its intrinsic bacterial properties, independent of immune cell involvement. This unique mechanism positions AUN as particularly valuable for treating immunosuppressive tumors [186]. Beyond direct oncolysis, complex metabolic interactions among members of the intratumoral microbiome can also activate host antitumor pathways. Studies have confirmed that *Peptostreptococcus anaerobius* and *Faecalibacterium prausnitzii* form an elaborate metabolic cross-feeding network in the gut: 7-dehydrocholesterol produced by *P. anaerobius* is converted to active vitamin D by *F. prausnitzii*, subsequently activating the host vitamin D receptor signaling pathway to suppress CRC development [187]. Regarding immunological regulation, specific components of the intratumoral microbiome can precisely modulate host immune responses through their metabolites. Research shows that indole-3-lactic acid, produced by *Lactobacillus reuteri* from tryptophan metabolism in the gut, targets the nuclear receptor RORγt to effectively inhibit TH17 cell differentiation, consequently downregulating the IL-17 signaling pathway and ultimately suppressing the development of CRC [183]. Collectively, these findings underscore the remarkable potential of the intratumoral microbiome as a novel strategic approach in cancer therapy, revealing multiple layers of microbial regulation that converge to inhibit tumor progression through complementary biological pathways.

#### Engineered bacteria

The concept of employing bacteria for cancer therapy is not new; it was first proposed in the nineteenth century when tumor regression was serendipitously observed in patients injected with Streptococcus pyogenes and Clostridium sporogenes [188] The fundamental principle remains exploitable, leveraging the intrinsic ability of certain bacteria to colonize hypoxic and immunosuppressive TME [189]. However, modern synthetic biology has transformed this crude concept into a sophisticated therapeutic platform. Contemporary genetic engineering enables precise augmentation of bacterial tumor-targeting. Strategies now extend beyond the display of adhesion peptides or tumor-associated antigens on the bacterial membrane. A key advancement is the design of complex synthetic gene circuits that integrate bacterial survival with specific tumor hallmarks, such as high lactate, low oxygen, and acidic pH [190, 191]. These circuits not only enhance tropism but also provide autonomous, localized control over bacterial proliferation and therapeutic payload release, thereby improving safety profiles. Furthermore, the utilization of quorum sensing (QS) systems allows for a coordinated, population-level behavior among therapeutic bacteria, enabling timed drug release only when a critical bacterial density is reached within the tumor, thus maximizing efficacy and minimizing off-target effects.

A wide array of bacteria, including *Salmonella*, *Escherichia coli* (e.g., the engineered strain EcN) [192], *Clostridium*, and *Bifidobacterium*, serve as versatile vectors [193–195]. Through genetic engineering, these chassis organisms can be programmed to encode and deliver a diverse repertoire of therapeutic payloads. Moving beyond traditional toxins and prodrug-converting enzymes, the frontier now includes the delivery of sophisticated molecules such as small interfering RNAs (siRNAs) for gene silencing, nanobodies against immune checkpoints, and engineered cytokines. The role of engineered bacteria in orchestrating anti-tumor immunity represents a paradigm shift. They can be designed as in situ bio-factories to continuously produce and deliver tumor antigens, ICIs and chemotactic cytokines [196]. This direct, localized delivery efficiently recruits and activates immune cells, effectively reshaping the "cold" immunosuppressive TME into a "hot" immunogenic one. Recent studies with strains such as PROT3EcT [197, 198] demonstrate advanced strategies for facilitating direct cytosolic drug delivery into tumor cells, overcoming a significant barrier in effective treatment.

The future of engineered bacteria lies in increasing sophistication, personalization, and integration. Moving beyond simple drug delivery vehicles, the next generation of microbial therapeutics will be programmable, autonomous systems capable of complex decision-making within the TME. Future progress will be driven by cutting-edge synthetic biology tools. CRISPR-based gene editing will allow for more precise and stable genomic integration of complex circuits. The development of multi-input logic-gated circuits is a key trend, enabling bacteria to respond only to a specific combination of tumor biomarkers (e.g., hypoxia AND high lactate AND low pH) [199, 200], drastically improving tumor-specificity and safety. For instance, a bacterium could be engineered to express a therapeutic payload only when a transcriptional activator for hypoxia is active AND a lactate-sensing riboswitch is triggered. Furthermore, the use of synthetic quorum sensing molecules orthogonal to natural bacterial communication will prevent cross-talk with resident microbiota and allow for precise, engineered population control. The convergence of diagnostics and therapy will mature into fully functional "theranostic" platforms. These systems employ sense-and-response circuits that detect intra-tumoral signals and dynamically modulate treatment. A prime example is an engineered *E. coli* strain designed to detect the high extracellular ATP concentrations often found in tumors. Upon sensing ATP, a bacterial genetic circuit is activated, leading to the production and release of a nanobody that blocks CD47 on tumor cells, thereby exposing them to phagocytosis by macrophages. This creates a closed-loop feedback system where the therapeutic action is directly controlled by the tumor's physiological state. The synergy between engineered bacteria and established therapies will be a dominant trend. Bacteria can be armed to express enzymes that convert systemically administered prodrugs into active chemotherapeutics specifically within the TME, thereby localizing toxicity. In immunotherapy, strains engineered to continuously secrete anti-CD47 nanobodies or CXCL16 chemokines can remodel the TME and synergize with ICIs to overcome resistance. A promising direction is metabolic niche engineering, where bacteria are programmed to consume immunosuppressive metabolites (e.g., lactate, adenosine) or produce immunostimulatory ones (e.g., L-arginine), directly shifting the immune context of the tumor towards a pro-inflammatory state.

### Synergy with tumor immunotherapy and clinical translation

In recent years, the field of cancer immunotherapy has developed rapidly, resulting in numerous significant breakthroughs. Among these advancements, the role of intra-tumoral microbiota has emerged as a novel therapeutic approach, intersecting with cancer immunotherapy in various ways. Clinical trials focusing on microbiota-based interventions have also begun to unfold. (Table 4). The close relationship between intra-tumoral microbiota and immunotherapy is primarily reflected in the influence of various microbial metabolites on immune cells and immune-related factors. For instance, Winnie Fong et al. [201] discovered that metabolites derived from *Lactobacillus gallinarum* can inhibit Treg cells by modulating the IDO1/Kyn/AHR axis, thereby enhancing the efficacy of anti-PD-1 therapy in CRC. Similarly, Joon Seok Park [202] found that blocking the PD-L2-RGMb interaction can overcome the microbial dependency resistance to PD-1 pathway inhibitors. The obstruction of the PD-L2-RGMb pathway mediated by antibodies, or the conditional deletion of RGMb in T cells, in conjunction with anti-PD-1 or anti-PD-L1 antibodies, promotes anti-tumor responses in various mouse tumor models that are otherwise unresponsive to these treatments. Additionally, Jiang SS [203] demonstrated that succinate derived from *Fusobacterium* can induce resistance to immunotherapy in CRC. Evidently, research exploring the intersection of microbiota and immune checkpoint therapies has been steadily increasing over the past two years, with a deeper understanding of the underlying mechanisms. This positions microbiota and their metabolites as promising new targets in cancer immunotherapy.

**Table 4:** Clinical trials related to intratumoral microbiome in cancer

Bactria	Objective	Data	NCT number
*E. coli* Nissle	Does treatment with ciprofloxacin followed by EcN for seven weeks affect disease activity in ulcerative colitis patients compared to the placebo control group	2011/05–2013/08	NCT01772615
*E. coli Nissle*	When using a combination of fluorouracil with other chemotherapy drugs (FLO, FOLFOX, FOLFOX-Bev, FOLFIRI), it is important to study whether the suspension of Escherichia coli Nissle has an effect on the duration and intensity of chemotherapy-induced diarrhea	2019/11-2021/05	NCT02706184
*VNP20009*	Phase I trial evaluating the efficacy in treating patients with advanced solid tumors	2000/05–2000/08	NCT00006254
*VNP20009*	This Phase I trial aims to evaluate the safety and toxicity of VNP20009, as well as its impact on tumor growth in patients with advanced or metastatic cancer (cancer that has spread from the primary site)	2018/07–2024/10	NCT00004988
*S.typhimurium*	Oral administration of the attenuated Salmonella typhimurium strain is safe and effective in patients with unresectable liver metastases from solid tumors	2010/04 2014/06	NCT01099631
*Clostridium Novyi-NT*	Determine the maximum tolerated dose of the bacterial therapy (Clostridium Novyi-NT) in the treatment of cancer	2018/07–2024/10	NCT03435952
*Clostridium Novyi-NT*	Measure the anti-tumor activity of C. novyi NT therapy in patients with refractory solid tumors by assessing the response within the tumor after treatment	2013/10–2017/10	NCT01924689
*Clostridium Novyi-NT*	Evaluate the safety of intravenous injection of Bacillus Calmette-Guerin (BCG) spores in patients with refractory solid tumors	2011/04–2013/06	NCT01118819
*Clostridium Novyi-NT*	A single intravenous (IV) infusion of C.novyi NT spores is administered to treat solid tumors that are non-responsive to standard treatment	2006/07-2008/09	NCT00358397

## Challenges and future perspectives

The distribution of intratumoral microbiome varies in terms of tissue origin, organ composition, and tissue localization, potentially linking it closely to the gut microbiota. Intratumoral microbiome plays a crucial role in regulating tumor progression and treatment efficacy. Moreover, the strategic use of microbiota presents a novel therapeutic approach, diagnostic and prognostic tool, and potential target for cancer treatment. It is noteworthy that intratumoral microbiome can modulate the immune microenvironment by promoting inflammatory responses or inhibiting anti-tumor effects, thus influencing tumor outcomes. The impact of intratumoral microbiome on anti-tumor immunity depends on its composition, interactions with cancer cells, and the state of the cancer itself.

In this review, we delve into the role of intratumoral microbiome in CRC, providing a detailed summary of the mechanisms by which intratumoral microbiome influence the onset and progression of CRC. We found that intratumoral microbiome activate oncogenic pathways, leading to the development of CRC, through mechanisms such as DNA damage and chronic inflammation. Due to their unique localization within the tumor, the strength of their effects and their destructive impact on the host are often greater than that of gut microbiota. Additionally, intratumoral microbiome, along with gut microbiota, secrete various metabolic products that influence the development of CRC through pathways such as ROS, STAT, and STING, as well as by modulating the immune microenvironment.

As a novel research subject, intratumoral microbiome is increasingly recognized and valued. Recent clinical trials targeting intratumoral microbiome have gradually demonstrated the feasibility and effectiveness of intratumoral microbiome as a clinical medical approach. This paper emphasizes the diversity of intratumoral microbiome in cancer treatment, whether as a significant biomarker indicating tumor occurrence and metastasis, as engineered bacteria capable of carrying difficult-to-administer drugs, or as a direct therapeutic strategy: eliminating harmful microbiota or supplementing beneficial microbiota within the tumor, all of which showcase the potential of intratumoral microbiome in cancer treatment.

The understanding and research of intratumoral microbiome remain superficial. We believe that intratumoral microbiome may show the following therapeutic and research potential in the future:

1. Interaction between Intratumoral and Gut Microbiota: Current research shows that intratumoral microbiome collaborates with gut microbiota in the occurrence and progression of cancer, particularly in influencing the TME, and can, to some extent, transform into each other. We consider exploring the heterogeneity and correlation between gut and intratumoral microbiome as a new direction in cancer treatment, and a deeper investigation into their complex roles in the immune microenvironment is crucial for cancer therapy.
2. Low Biomass but High-Intensity Effects: Intratumoral microbiome have a low biomass in the body but often exert stronger effects. Investigating their specific mechanisms of action in tumor development and progression may lead to more effective cancer treatment strategies, achieving better therapeutic outcomes with lower dosages.
3. Engineered Bacteria and Bacterial Drug Delivery Systems: Research on engineered bacteria and bacterial drug delivery systems represents another pathway for intratumoral microbiome to participate in cancer treatment. More advanced ex vivo editing technologies may find a better balance between bacteria and the host for the intervention of cancer treatment.
4. The Pivotal Role of AI: The complexity and high-dimensionality of intratumoral microbiome data necessitate the integration of AI to unlock its full diagnostic, prognostic, and therapeutic potential. AI-powered predictive models can analyze a patient's unique intratumoral microbiome profile, tumor genetics, and immune contexture to identify the most promising therapeutic approach. For instance, AI can help determine whether a patient is likely to benefit from targeted pathogen depletion, administration of a beneficial consortium, or a specific engineered bacterial strain. Furthermore, AI is instrumental in the design phase of engineered microbes, capable of optimizing complex synthetic gene circuits for controlled therapeutic payload delivery in response to specific tumor microenvironment cues. By modeling bacterial population dynamics and quorum sensing behaviors, AI can help design safer and more effective microbial robots that maximize antitumor efficacy while minimizing off-target effects. Finally, machine learning algorithms are exceptionally suited to decipher the complex, non-linear relationships within the microbiome, integrating microbial abundances, spatial distribution, and host clinicopathological data to build superior diagnostic and prognostic models that overcome the challenge of tumor heterogeneity.

In recent years, research on intratumoral microbiome has garnered significant attention and progress, with numerous clinical trials currently underway. However, current studies are limited. The mechanisms by which intratumoral microbiome affect anti-tumor immunity and treatment efficacy are not yet fully understood, impeding the clinical application of microbiota-related therapeutic strategies in cancer. Therefore, extensive validation through preclinical models and clinical trials is necessary. The integration of AI, as a powerful analytical and predictive tool, will be essential to accelerate this validation process and decipher the underlying mechanisms. We believe that effective tumor treatment can be achieved in the future through the management or targeting of microbiota, potentially in combination with immunotherapy, and guided by AI-driven insights.

## Data Availability

Not applicable.
